# A case of nontraumatic acetabular fracture during epileptic seizure in an elderly patient: Imaging diagnosis and endovascular treatment

**DOI:** 10.1016/j.radcr.2025.10.040

**Published:** 2025-11-15

**Authors:** Noriko Ito, Mitsuru Matsuki, Akihiro Nakamata, Sota Masuoka, Emiko Chiba, Kohei Hamamoto, Hiroyuki Fujii, Harushi Mori

**Affiliations:** a Department of Diagnostic Radiology, Jichi Medical University Hospital, 3311-1 Yakushiji, Shimotsuke-shi, Tochigi-ken, 329-0498, Japan; b Department of Pediatric Radiology, Jichi Children's Medical Center Tochigi, 3311-1 Yakushiji, Shimotsuke-shi, Tochigi-ken, 329-0498, Japan

**Keywords:** Nontraumatic fracture, Acetabular fracture, Epileptic seizure, Transcatheter arterial embolization

## Abstract

Acetabular fractures are usually caused by high-energy trauma, and seizure-induced cases resulting from strong muscle contractions are extremely rare. We report the case of a 90-year-old man with osteoporosis who incurred a nontraumatic acetabular fracture during a seizure. Computed tomography revealed an internal iliac artery injury, which was promptly treated with transcatheter arterial embolization. As such vascular injuries, though rare, require early diagnosis and treatment, radiologists must be aware of the associated imaging findings and management, particularly in cognitively impaired patients.

## Introduction

Acetabular fractures commonly have a traumatic etiology, resulting from high-energy mechanisms such as motor vehicle accidents or falls from significant heights [1]. Nontraumatic acetabular fractures due to seizures are extremely rare [2]. The proposed mechanism involves the sudden, simultaneous contraction of multiple large muscle groups [[2], [3], [4], [5]].

Traumatic pelvic fractures can be fatal if vessels such as the internal iliac artery are injured, necessitating early detection and prompt intervention [6]. Although vascular injury caused by nontraumatic fractures following seizures are rare, such injuries can prove fatal [6]. Radiologists should therefore be familiar with the associated imaging features and appropriate management of nontraumatic acetabular fractures.

## Case report

**Fig. 1:**
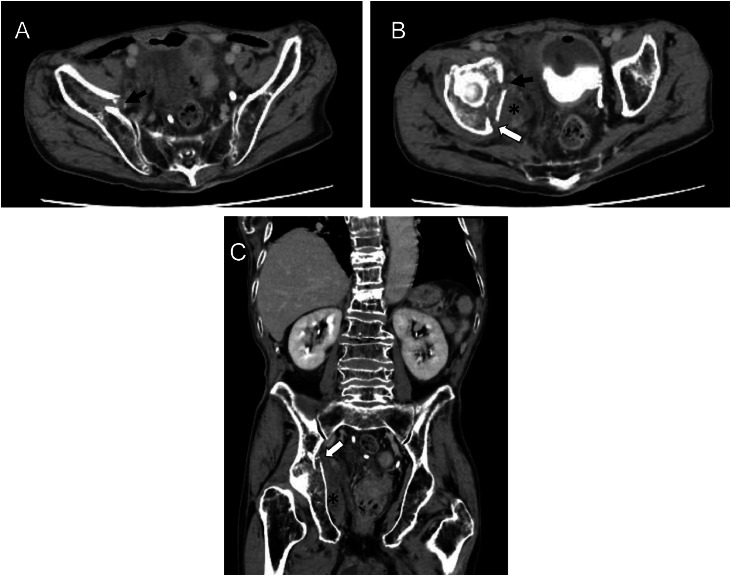
Contrast-enhanced CT for pulmonary embolism and deep vein thrombosis screening, A–C) Medial fracture involving both the anterior (A, B: black arrows) and posterior columns (B, C: white arrows) of the right acetabulum, along with swelling and hyperdensity of the right iliacus muscle (B, C: asterisks), suggest vascular injury and disruption caused by right acetabular fractures incurred during epileptic seizure. On the coronal reformatted image (C), multiple compression fractures of the thoracolumbar vertebral bodies are apparent, and the trabecular architecture appears diffusely coarsened, suggestive of osteoporotic changes.

**Fig. 2:**
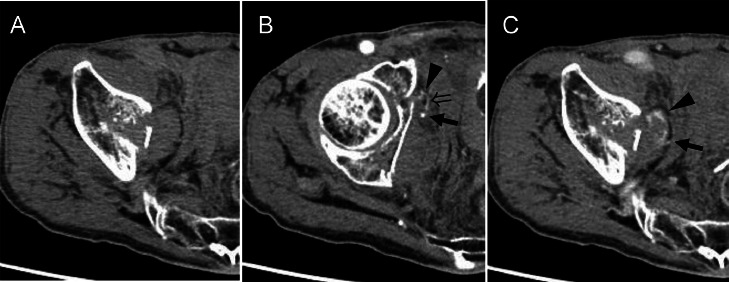
Dynamic contrast-enhanced CT. (A) Noncontrast phase; (B) arterial phase; and (C) equilibrium phase. Compared to the noncontrast phase (A), contrast extravasation (arrowheads) is shown in both the arterial (B) and equilibrium (C) phases. A branch (open arrow) of the right obturator artery (arrow) is situated adjacent to the site of extravasation, suggesting injury or disruption to this arterial branch as the likely source of hemorrhage.

**Fig. 3:**
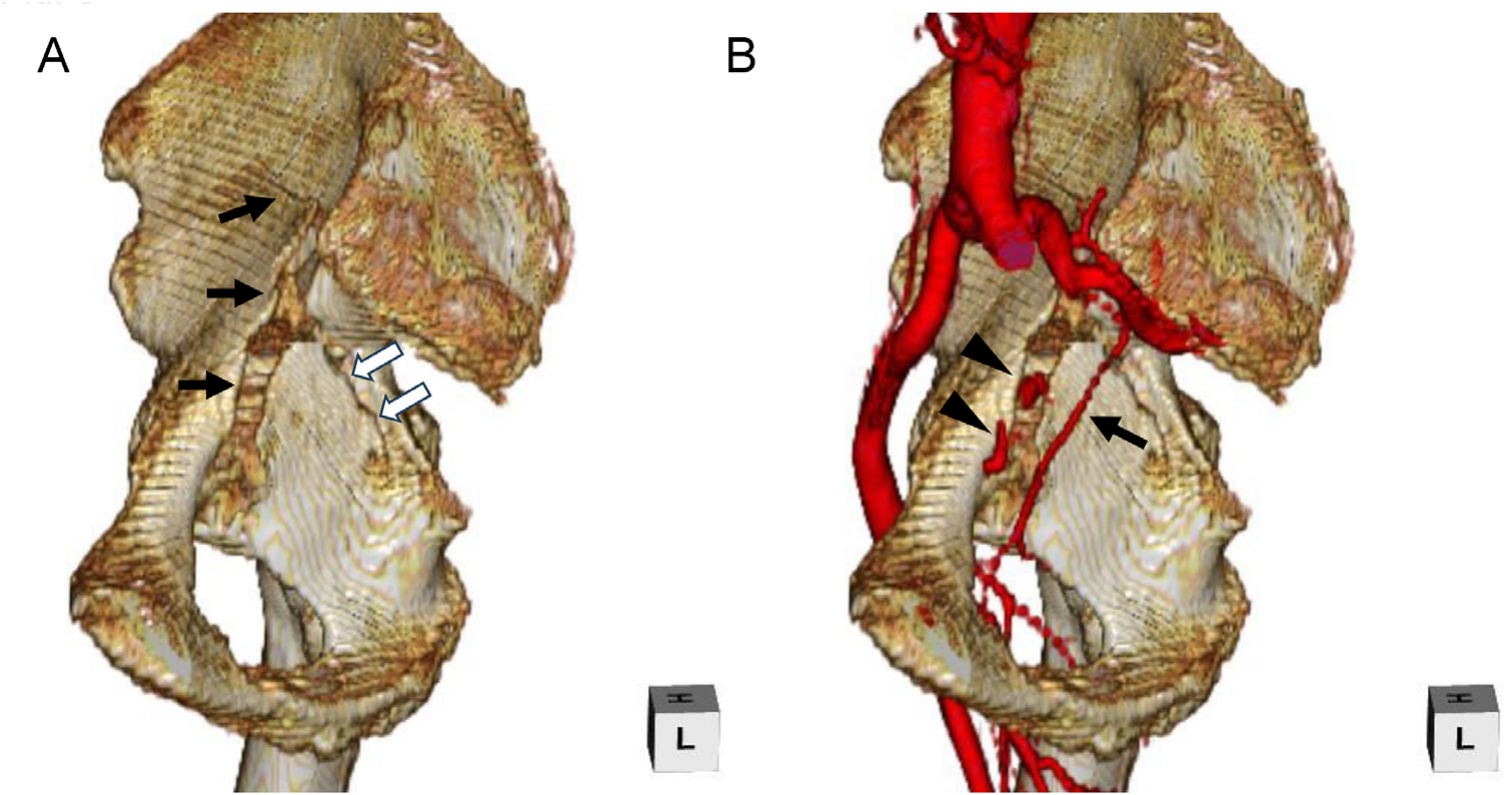
Three-dimensional (3D) volume-rendered image. The 3D volume-rendered image of the right acetabulum (A) clearly and spatially demonstrates a medial wall fracture involving both the anterior (black arrows) and posterior (white arrows) columns, with medial displacement of the fracture fragment. This pattern may have resulted from repetitive loading of the femoral head against the acetabulum, in combination with contraction of the medial periarticular muscles during recurrent seizures. A fusion image of the 3D volume-rendered right acetabulum and the right iliac artery (B) demonstrates the spatial relationship among the fracture site of the right acetabulum, the right obturator artery (arrow), and the site of contrast extravasation (arrowheads).

A 90-year-old man was a resident of a nursing home. For the past 3 weeks, he had been unable to take in food or fluids and had been receiving intravenous fluids at a local clinic. A caregiver discovered him in the midst of a generalized seizure on his bed in his room, without any evidence of trauma. The seizure lasted for more than 20 minutes. He was subsequently transported to our hospital by emergency services. The patient had a medical history of cerebral infarction, dementia, hypertension, and paroxysmal atrial fibrillation. The family history remains unknown. Activities of daily living (ADL) were originally low, and Glasgow Coma Scale (GCS) score was 15 (E4V5M6). Emergency computed tomography (CT) of the head revealed chronic cerebral infarction. On admission, the patient presented with stable blood pressure, but with tachycardia and tachypnea. Laboratory tests revealed mild anemia. The D-dimer level was elevated, at 19.1 µg/mL (reference, <1 µg/mL). Contrast-enhanced CT was performed to evaluate for pulmonary embolism and deep vein thrombosis. No evidence of thrombosis was found, but a medial fracture involving both the anterior and posterior columns of the right acetabulum, along with swelling and hyperdensity of the right iliacus muscle, were detected incidentally (Fig. 1). Based on these findings, the diagnosis was nontraumatic acetabular fracture incurred during a seizure, complicated by vascular injury. Subsequent dynamic contrast-enhanced CT revealed contrast extravasation within the right iliacus muscle, and injury to branches of the right obturator artery was suspected as the bleeding source (Fig. 2, Fig. 3).

**Fig. 4:**
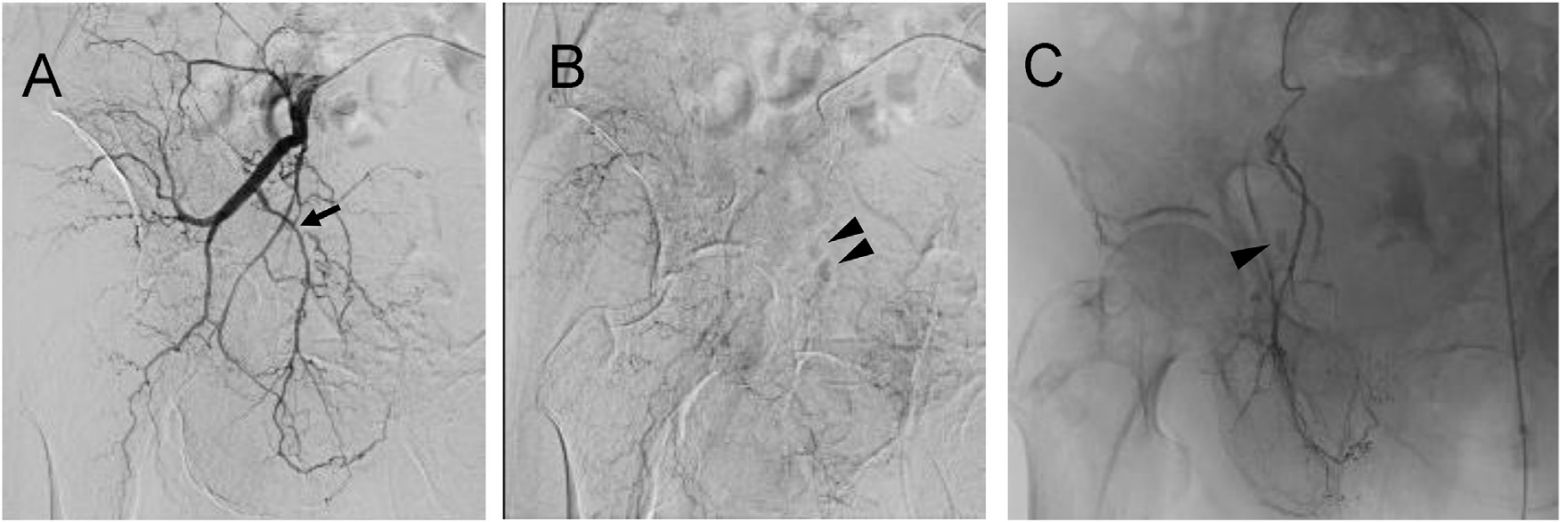
Right internal iliac artery angiography (A) Arterial phase. (B) Delayed phase. (C) Selective angiography of the obturator artery. Angiography of the right internal iliac artery (A, B) demonstrates contrast extravasation (B, arrowheads) in the delayed phase, presumably originating from a branch of the right obturator artery (A, arrow). Selective angiography of the obturator artery (C) shows extravasation from the branches (arrowhead). Embolization of the right obturator artery is performed using a gelatin sponge, achieving successful hemostasis.

Emergency angiography was performed for hemostasis. Angiography of the right internal iliac artery revealed contrast extravasation in the peripheral branch of the right obturator artery (Fig. 4A and B). Selective angiography using a microcatheter confirmed extravasation from branches of the right obturator artery (Fig. 4C), which was therefore embolized using gelatin sponge. Final angiograms of the internal and common iliac arteries confirmed cessation of contrast extravasation.

After treatment, the patient showed no decrease in blood pressure or progression of anemia, and conservative treatment was provided with indirect traction on the right lower leg. As the condition of the patient was stabilized, he was transferred to a nearby hospital on the day 12 of hospitalization.

## Discussion

Acetabular fractures can be classified into traumatic and nontraumatic types, with the majority being traumatic in nature and typically associated with high-energy mechanisms such as motor vehicle accidents or falls from height [1]. Traumatic fractures may occur when axial force is transmitted through the femur to the acetabulum via the flexed knee, as seen in dashboard injuries, or during vertical loading from landing on the feet after a fall from height [7]. In contrast, nontraumatic acetabular fractures are rare but have been associated with seizures or epileptic episodes. The underlying mechanism is believed to involve intense muscle contractions during seizures, generating substantial force across the joints [3]. Nontraumatic fractures incurred during seizures most commonly involve the proximal limbs, shoulders, spine, and pelvis, and involvement of the acetabulum is exceedingly uncommon [3,8,9]. In cases of nontraumatic acetabular fracture during seizures, forceful contractions of the periarticular hip muscles may drive the femoral head medially and proximally, resulting in fracture of the medial acetabular wall and, in some instances, fracture-dislocation of the hip with medialization of the femoral head [2,4,5]. In nontraumatic fractures caused by seizures, combined fractures of both the anterior and posterior columns are reported as the most common pattern [10]. In the present case, consistent with previous reports, medial wall fracture of the right acetabulum involving both the anterior and posterior columns was observed. Further, the fracture fragments were displaced medially. This may have resulted from repetitive loading of the femoral head against the acetabulum, combined with contraction of the medial periarticular muscles during recurrent seizures [6,10]. These characteristic fractures were visualized on CT, and 3-dimensional, volume-rendered CT provided detailed spatial representation, which was useful for understanding the fracture pathology and its anatomical relationship to hemorrhagic sources.

Risk factors for nontraumatic fractures include advanced age, osteoporosis, osteomalacia, a muscular build, and certain medications such as corticosteroids and antiepileptic drugs [9,[11], [12], [13]]. In the present case, advanced age and osteoporosis were considered to have contributed to the fracture.

Treatment for acetabular fractures typically involves conservative management when joint congruency is preserved and displacement is minimal (usually less than 2 mm). However, if hip joint congruency is compromised or the fracture is significantly displaced, surgical fixation is the standard treatment.

Nontraumatic acetabular fractures due to seizures, particularly when bilateral, are associated with a poor prognosis, with a reported mortality rate of 18.5% [6]. Contributing factors include hemorrhage due to vascular injury and sepsis resulting from sacral ulcers secondary to prolonged immobilization [4,6]. Although no reports to date have documented the use of transcatheter arterial embolization for managing hemorrhage secondary to vascular injury in nontraumatic acetabular fractures developing during seizures, this treatment option should be recognized as potentially effective, similar to its established role in traumatic fractures.

In conclusion, nontraumatic acetabular fractures occurring during seizures in elderly patients are at risk of being overlooked in clinical practice. Radiologists should be familiar with the imaging features and be able to contribute actively to appropriate management.

## Patient consent

Written informed consent was obtained from the patient.

## References

[1] Stevens J.M., Shiels S., Whitehouse M.R., Ward A.J., Chesser T.J., Acharya M. (2019). Bilateral acetabular fractures: mechanism, fracture patterns and associated injuries. J Orthop.

[2] Heyer J.H., Thakkar S.C., Zittel K., Tozzi J.E. (2020). Bilateral acetabular fractures treated with delayed total hip arthroplasty. Arthroplast Today.

[3] Gill J.R., Murphy C.G., Quansah B., Carrothers A.D. (2015). Seizure induced polytrauma; not just posterior dislocation of the shoulder. BMJ Case Rep.

[4] Granhed H.P., Karladani A. (1997). Bilateral acetabular fracture as a result of epileptic seizure: a report of two cases. Injury.

[5] Takahashi Y., Ohnishi H., Oda K., Nakamura T. (2007). Bilateral acetabular fractures secondary to a seizure attack caused by antibiotic medicine. J Orthop Sci.

[6] Nehme A.H., Matta J.F., Boughannam A.G., Jabbour F.C., Imad J., Moucharafieh R. (2012). Literature review and clinical presentation of bilateral acetabular fractures secondary to seizure attacks. Case Rep Orthop.

[7] Letournel E., Judet R., Elson R. (1993).

[8] Finelli P.F., Cardi J.K. (1989). Seizure as a cause of fracture. Neurology.

[9] Grzonka P., Rybitschka A., De Marchis G.M., Marsch S., Sutter R. (2019). Bone fractures from generalized convulsive seizures and status epilepticus: a systematic review. Epilepsia.

[10] Mousafeiris V.K., Vasilopoulou A., Chloros G.D., Panteli M., Giannoudis P.V. (2022). Management and outcomes of bilateral acetabular fractures: a critical review of the literature. Indian J Orthop.

[11] Yngstrom K., Stapleton E., Aberman Z., Galos D. (2020). Bilateral acetabular fractures associated with seizures: a report of 2 cases. JBJS Case Connect.

[12] Nilsson O.S., Lindholm T.S., Elmstedt E., Lindbäck A., Lindholm T.C. (1986). Fracture incidence and bone disease in epileptics receiving long-term anticonvulsant drug treatment. Arch Orthop Trauma Surg (1978).

[13] Zhang Q., Gao F., Sun W., Li Z. (2021). Bilateral multiple periprosthetic hip fractures and joint dislocations secondary to general convulsive seizures. BMC Musculoskelet Disord.

